# A systematic review of qualitative research of eating disorders in autistic adults: convergent and divergent experiences

**DOI:** 10.3389/fpsyt.2026.1953234

**Published:** 2026-10-15

**Authors:** Becky Watts, Samuel J. R. A. Chawner, Gareth Burchell, John R. E. Fox

**Affiliations:** 1 Clinical Psychology, School of Psychology, University of Sheffield, Sheffield, United Kingdom; 2 Division of Psychological Medicine and Clinical Neurosciences, Cardiff University, Cardiff, United Kingdom

**Keywords:** autism, eating disorders, lived experience, qualitative, synthesis

## Abstract

**Introduction:**

Autism and eating disorders frequently co-occur, yet relatively little is known about how autistic adults experience eating disorders.

**Methods:**

This systematic review synthesised qualitative evidence to explore the lived experiences of autistic adults with an eating disorder and inform autism-informed clinical practice. A systematic search identified 13 eligible studies, which were appraised using the Critical Appraisal Skills Programme (CASP) Qualitative Checklist and synthesised using thematic synthesis.

**Results:**

Findings were organised into three themes: eating disorder experiences cannot be understood independently of autistic ways of processing the world; recovery is enabled through developing self-understanding and integrating autism into personal identity; and recovery is facilitated when support aligns with autistic needs rather than neurotypical assumptions. Autistic characteristics, particularly sensory sensitivities, interoceptive differences, and a preference for predictability, fundamentally shaped the development, maintenance, and recovery of eating disorders. Sensory-driven food restriction was frequently experienced independently of body image concerns, highlighting potential conceptual overlap with Avoidant/Restrictive Food Intake Disorder (ARFID).

**Discussion:**

Autistic adults described eating disorders as inseparable from their autistic experience, indicating the need for a paradigm shift from conceptualising autism and eating disorders as co-occurring conditions towards a model rooted in lived phenomenological experience. Clinical assessment, formulation, and intervention should adopt autism-informed, individualised approaches that recognise sensory processing and interoceptive differences, while future research should examine these mechanisms across a broader range of eating disorder diagnoses.

**Systematic review registration:**

https://www.crd.york.ac.uk/PROSPERO/, identifier CRD420261375735.

## Introduction

Eating disorders are serious, yet treatable, mental health conditions characterised by persistent disturbances in eating behaviours that impair physical health or psychosocial functioning, often linked to an individual’s perceptions of weight, shape, and food control concerns (1, 2). More recent diagnoses also incorporate drivers such as food avoidance linked to sensory aversions and concern regarding aversive consequences of eating (3). According to the *Diagnostic and Statistical Manual of Mental Disorders* (5th ed., text rev.; DSM-5-TR), eating disorders include conditions such as anorexia nervosa (AN), bulimia nervosa (BN), binge-eating disorder (BED), avoidant/restrictive food intake disorder (ARFID), and other specified feeding or eating disorder (OSFED) (1, 4). At least 1.25 million people in the UK have experienced an eating disorder, with many often struggling in secret (4). In addition to their high prevalence, eating disorders, particularly anorexia nervosa, are associated with a markedly elevated mortality rate (5).

Autism is a neurodevelopmental condition characterised by differences in social communication, behavioural flexibility, and sensory processing (1, 6). It is worth noting that the behaviourally defined criteria outlined within the DSM-V continue to be “impairment” or “deficit” focussed and diagnostic terms use the language of “disorder”. Contrastingly, the neurodiversity paradigm is reshaping how we understand autism, how we conduct research, the language used and how autistic people are supported by conceptualising autism as reflecting natural variation in cognitive and perceptual styles rather than solely a deficit that has often dominated the narrative from diagnostic frameworks (7). Thus, within this review, we will be using the term autism, rather than the diagnostic term “autism spectrum disorder” outlined within the DSM-V. Identity first language and reference to autistic people will also be used as in English speaking countries the literature has highlighted a preference for this (8).

A number of traits commonly associated with autism may have relevance to eating behaviours. These include sensory sensitivities that can influence responses to food textures, tastes, and smells (9, 10), as well as differences in interoceptive awareness that may influence a person’s recognition of their internal hunger and satiety cues (11). A core feature of autism is a preference for routine and predictability, which may contribute to selective eating patterns, such as rigidity (12). In addition, social communication differences may shape experiences of eating within social contexts and interactions with healthcare services. While these traits vary widely, they highlight several important mechanisms through which autistic experiences may intersect with the development of eating-related difficulties.

Increasing evidence suggests a significant overlap between autism and eating disorders, with a significant focus on AN (13–15). Studies have identified elevated rates of autistic traits among individuals receiving treatment for eating disorders (13, 16), while autistic individuals self-report higher levels of disordered eating behaviours (17). This overlap is driven by shared traits including but not limited to cognitive rigidity, sensory sensitivities, and differences in interoceptive awareness (18–20). These characteristics may influence how eating disorders develop, are experienced, and are maintained in autistic individuals (14, 17). For example, sensory sensitivities may shape food preferences and aversions, while preferences for routine and predictability may reinforce rigid eating patterns (17, 19). Growing clinical awareness of this intersection has also highlighted challenges in diagnosis and treatment, with autistic individuals often experiencing delays in identification or difficulties engaging with conventional eating disorder interventions (15). Together, these findings suggest that the relationship between autism and eating disorders warrants further investigation, particularly in relation to how these conditions are experienced by autistic individuals themselves (14).

Despite growing recognition of the overlap between autism and eating disorders, several limitations remain within the existing literature (14, 15). The majority of research in this area has been quantitative in its methodological approach, focusing on prevalence estimates, symptom profiles, and neurocognitive traits (13, 18). While quantitative approaches have been valuable in demonstrating the existence of an overlap, their insight is limited into how eating disorders are experienced by autistic individuals themselves (14). A phenomenological approach allows researchers to understand the condition and associated behaviours, but also what experiences lie underneath them (21). Understanding the lived experience of autistic individuals with eating disorders is therefore essential for developing a more comprehensive understanding of how these conditions intersect (14, 15) and provide valuable insight into the ways individuals interpret, experience, and navigate eating-related difficulties within the context of autistic traits and social environments (17). Such perspectives can shine a light on aspects of eating disorders that may not be captured through diagnostic criteria or quantitative measures, including sensory experiences of food, the role of routine and predictability in eating behaviour, and interactions with healthcare services (15, 18, 19).

Although qualitative research exploring the lived experience of autistic individuals with eating disorders has increased in recent years, particularly in relation to anorexia nervosa and treatment experiences (14, 17, 22), the evidence base remains comparatively limited across the broader spectrum of eating disorder diagnoses. Contemporary reviews continue to highlight a relationship between autistic traits and eating disorder psychopathology, particularly within anorexia nervosa, including differences in cognitive style, rigidity, and sensory processing (17, 23). However, research has frequently focused on diagnostic categories or mixed perspectives from autistic individuals, carers, and clinicians rather than exclusively focusing on first-person accounts (14, 23). Consequently, autistic perspectives continue to be underrepresented within eating disorder research, with less emphasis on how autistic individuals themselves understand and make sense of eating-related difficulties in everyday life. Synthesising qualitative evidence therefore remains important to better understand how autistic traits shape the lived experience of eating disorders, including sensory sensitivities, emotional regulation differences, coping strategies, social experiences, perceived barriers within treatment settings, and the ways individuals navigate eating-related difficulties within the context of autistic traits and social environments (14, 17).

The present study therefore aims to conduct a systematic review of qualitative research exploring the lived experiences of autistic adults with eating disorders and disordered eating. By synthesising existing qualitative evidence, the review seeks to deepen understanding of how disordered eating is experienced within autistic populations and to move beyond diagnostic categories to consider traits that may reflect shared underlying mechanisms. Specifically, the review aims to: (1) characterise the lived experiences of autistic adults with eating disorders, including experiences and traits not fully captured by categorical diagnostic labels and (2) generate clinical recommendations that may inform more tailored, autism-informed approaches to assessment and intervention.

The review sought to answer the question: *How do autistic characteristics influence the lived experience, understanding and recovery of eating disorders in autistic adults*?

## Methodology

### Design

In order to synthesise the existing qualitative research exploring the lived experiences of autistic adults with eating disorders, we reported in accordance with the Preferred Reporting Items for Systematic Reviews and Meta-Analyses (PRISMA) guidelines (24).

A thematic synthesis approach (25), was used to analyse findings across the included studies, as it is well suited to the integration of qualitative research exploring lived experience. Thematic synthesis enabled the systematic coding of textual data and the development of both descriptive and higher-order analytical themes, allowing patterns to be identified across studies while preserving the depth and context of individual accounts (25). This approach is particularly appropriate for the present review, which seeks not only to capture the lived experiences of autistic adults with eating disorders but also to generate novel insights into underlying mechanisms and inform clinical recommendations. By moving beyond simply collecting the findings, thematic synthesis facilitates the development of interpretive insights that extend across studies (26).

### Search strategy

A systematic search of electronic databases was conducted to identify relevant studies. The following databases were searched: PsycINFO, MEDLINE, Scopus and CINAHL.

The search strategy combined terms relating to autism, eating disorders, and qualitative research using Boolean operators (AND/OR):

- (“autis*” OR “ASD” OR “ASC”).
- AND (“eating disorder*” OR “anorexia” OR “bulimia” OR “binge eating” OR “disordered eating” OR “arfid” OR “osfed”).
- AND (qualitative OR interview* OR “lived experience” OR thematic OR phenomenolog * OR ethnograph* OR “grounded theory” OR narrative* OR “focus group*” OR “case stud*” OR “content analysis” OR “discourse analysis”).
- AND (adult*).

Searches were limited to peer-reviewed articles published in English. Reference lists of included studies were hand-searched to identify additional relevant articles.

### Eligibility criteria

To ensure a rigorous approach to study selection, the eligibility criteria for this systematic review were structured using the SPIDER (Sample, Phenomenon of Interest, Design, Evaluation, Research type) framework (27). While traditional systematic reviews often utilise the PICO framework, SPIDER is uniquely optimised for qualitative and mixed-methods research, as it prioritises the exploration of subjective experiences rather than quantitative outcomes (27). The application of the SPIDER components to this review’s research question is detailed in **Table 1** below.

**Table 1:** SPIDER framework.

Element	Application to current review
S - Sample	Studies involving autistic adults (≥18 years), either formally diagnosed or self-identified
PI – Phenomenon of Interest	Primary data from autistic adults with lived experience of eating disorders or disordered eating
D - Design	Studies employing quantitative methodologies e.g semi structured interviews, qualitative surveys, autoethnographic accounts
E - Evaluation	Participant perspectives, barriers to care, coping mechanisms or views on recovery
R – Research type	Qualitative research or mixed-methods studies containing distinct qualitative data. Published in peer reviewed journals and in English.

### Exclusion criteria

- Quantitative-only studies.
- Studies focusing exclusively on children or adolescents.
- Perspectives of parents, carers or professionals only.
- Reviews, editorials, dissertations, or grey literature.

### Study selection

All identified records were imported into reference management software and duplicates removed. Study selection was conducted in two stages:

1. Title and abstract screening to identify potentially relevant studies.
2. Full-text screening against the eligibility criteria.

The selection process is documented and presented using a PRISMA flow diagram. A second reviewer independently screened a subset of studies to enhance reliability.

### Data extraction

Data was extracted using a data extraction form, seen in **Table 2**. The following information was collected:

**Table 2:** Data extraction form for included studies.

Authors and year of publication	Data collection and analysis methods	Key themes	Participant quotes
(Babb, C., Brede, J., Jones, C. R., Elliott, M., Zanker, C., Tchanturia, K.,… & Fox, J. R. (28)). ‘It’s not that they don’t want to access the support … it’s the impact of the autism’: The experience of eating disorder services from the perspective of autistic women, parents and healthcare professionals. *Autism*, *25*(5), 1409-1421.	Interview Schedule. Identified via thematic analysis	Misunderstanding autism and autistic traits One treatment does not fit all Improving accessibility and engagement within Services	When I was in hospital, I kept getting told off for walking on tip toes and for fidgeting a lot … they thought I was doing these things to burn more calories, except I’d been doing them for as long as I could remember. I felt … very criticised. I was being very resistant – or labelled as being very resistant. I don’t think they took into account how … difficult I found it seeing lots of different people. It felt like they didn’t care … I used to like to sit in the same seat, just because I knew where it was, and every day a different staff member would go and sit there. And they wouldn’t understand why I was getting upset. When I explained about needing the food separate, they were very good about accommodating that. And they would separate out the food, and they didn’t necessarily challenge that … but they also gave me the opportunity to try it differently if I wanted to. With the CBT, maybe the problem is that they assume you have a lot of these skills already … Therefore, I couldn’t even begin to make changes, because I don’t have any of those foundational skills. I didn’t realise anybody would need me to. I wasn’t realising that I was being taught a general technique rather than to alleviate a particular problem. [CBT] helped me to understand why I thought and felt the way I did … that understanding helps you to kind of … restructure your thoughts and made me realise what wasn’t true. I’m largely silent in any sort of group situation because I don’t know what’s expected of me. I’m worried of saying the wrong thing or I worry about misinterpreting people. They had other groups as well, one which was just a general talking group, which I found was really unhelpful because they had no structure to it. DBT was really helpful … we spent a long, long time talking through emotions, identifying them and trying to think about how to regulate, how to manage them … DBT was about [emotion regulation] skills, and that was really important. Emotion regulation was really important to me … when I was in distress, I would just self-harm, just become very unwell. But after that, I actually started to think what was going on … I think that’s what really helped to improve my eating at the time. [The occupational therapist] helped me get a volunteer placement at my old primary school … that was the best therapy I ever got, because it was practical and gave me something to do. She basically didn’t believe me when I said this is not about [body image] … she wanted to do body image therapies with me and I … found it humiliating, and also irritating and condescending that I was being not listened to. We didn’t really focus on my eating … They made me focus on emotion regulation, and just mindfulness really, and social aspects, and that really helped me. She understood how best to communicate with me, so she gave me written summaries after my appointments, she gave me written information and we used goal setting to help plan my care. Although I found the groups more challenging, I knew what I had to do during my day. I found unstructured time difficult, and I used to spend my time making my own structure. The adjustments they agreed were … agreeing a time and day for key work sessions, giving me plenty of notice of change … and I could bring my soft duvet cover in and that then meant that I was comfortable and I could sleep and it’s just minor things like that that can make a really big difference.
(Brede, J., Babb, C., Jones, C., Elliott, M., Zanker, C., Tchanturia, K.,… & Mandy, W. (17)). “For me, the anorexia is just a symptom, and the cause is the autism”: Investigating restrictive eating disorders in autistic women. *Journal of autism and developmental disorders*, *50*(12), 4280-4296.	Semi structured interviews Identified via thematic analysis	Sensory sensitivities Social interaction and relationships Self and identity Difficulties with emotions Thinking styles Need for control and predictability	The fact it’s all mixed up, which in my head it shouldn’t all be mixed up, so for me a salad is actually a terrifying food!”. “That feeling of putting on weight … that’s what kind of sends me back into restricting food, because it’s not about ‘oh god my stomach looks really big’, it’s more about ‘I don’t like the sensation of how my stomach feels’”. “I’m not very good at judging my own emotions or physical sensations. I don’t really fully understand my thirst and hunger responses, or my fullness responses, so that really influences my eating because I can binge or miss meals very, very easily”. “I think I was lonely a lot after [my only friend changed school] and that affected it, and I could just get engrossed in food and exercise and just forget about everything else”. “The moment when I stopped eating at school, was because there was a big canteen, lots of people, lots of social stuff going on, lots of noise”. You constantly feel like you’re failing, you constantly feel different, you think it’s all your fault because you don’t know that there is something different about you”. “I have never had much of a sense of self, and I think possibly [AN] then became a little bit like an identity. Going into hospital and being aware that everybody has the same condition, you then do become a lot more aware of some of the anorexia traits and you do sort of take them on”. It was more like behaviours that I engaged with to feel calm, but would lead to catastrophic weight loss”. “When I was restricting my eating, I would get this feeling of just calmness, and I know that I am safer, I know that I am not going to experience these meltdowns that made me feel embarrassed and frightened.[ … ] So I was no longer just losing it”. “My OCD [obsessive compulsive disorder] started to get worse as I started to fight my eating disorder. I just seem to have kind of variations on the same theme, with the OCD and with the eating disorder. The problem seemed to be not what the content of my thoughts was, but how I thought”. “I misinterpret [emotions] as physical symptoms and I get very anxious about it: Am I unwell? Am I going to vomit? And that’s when I stop eating because I know that will dampen things down and calm them, so my emotions are feeding into my eating disorder behaviours, whereas I think my difficulties in perhaps coping with emotions stem perhaps more from the autism”. “I sometimes imagine life without [AN], and then think, well actually, I would still have a lot of problems, but I wouldn’t have my coping mechanism”. “The autism and the routine and rigid thinking maintained the eating disorder. I think that’s why my recovery has taken so long for me to get to what I would call true recovery. For me, the anorexia is just a symptom, and the cause is the autism”. “Sometimes it’s just habit that I will [engage in ED behaviours], because that’s what I have done for the last 15 years, rather than a driven behaviour, “I seem to have a strong need for control; I will always try and fill it with something. And if I could get rid of that, if I could learn to think differently … that would probably be the only way I could really recover”.
(Creese, M., Hampton, S., Brede, J., Babb, C., Elliott, M., Serpell, L.,… & Mandy, W. (29)). “From that moment, everything has changed”: The experience of women with anorexia nervosa receiving a diagnosis of autism. *European Eating Disorders Review*, *33*(4), 825-834	Reflexive thematic analysis on secondary data of 17 semi structured interviews	The search for understanding and support A shifting moment Taking control	‘I’ve always felt like I’m on the outside looking in’. …it also made me feel less alone because then I realised that here are other people who also have both [ED and autism], and other people have similar experiences. I don’t think people reacted in a good way particularly … I think people misbelieve me, they see me as so functional and they see how much I’m able to achieve, but they don’t see how much pedalling is going on beneath the surface…. And I have never had a proper psychiatric assessment outside of an ED setting. they previously diagnosed me with borderline personality disorder but I didn’t relate to it at all’. So, I seemed to collect a whole set of mental health diagnoses…. ‘They didn’t seem [to be] a very clear definition of the boundaries of the [autism] diagnostic criteria they applied to females’. If only I’d known different, I could have possibly managed this with some adaptions, I could have possibly done that, I possibly wouldn’t have been so hard on myself on achieving things. I think I had years of struggling with my mental health and anorexia and I reached a point where I was just, I was completely burned out, and I had, I suppose a breakdown. After that point I had to stop work. …They spent about half a day with me and at the end of the assessment they suggested I had ASD. …what’s wrong with me because I’m just going mad. I can’t fit in, I can’t get on with people, I can’t hold down a job, so if it’s not what everybody’s saying it is [AN] or what it could be, what on earth is it? I was starting to think I had a brain tumour and all sorts of things because I couldn’t cope. So you have an Asperger’s service, an autism service around if you’re lucky, an ED service around if you’re lucky. But … There doesn’t seem to be a kind of merging of them. I go to the autism service, and even going there I find really difficult. I feel like I don’t even fit in. Because I feel, we work on the autism, but we also work on the ED as well, so it is like a more holistic, I suppose, and that has been really effective. ‘from that moment everything has changed’. There were a lot of differences that weren’t explained by the anorexia. And we would try constantly “there is something else, there is something else”. And after all these years, I finally found what this something else was. I now understand that all these things came on the foundation of the autism. I’ve always felt different and like I didn’t fit in and it was kind of like oh actually that’s because I am different and I don’t particularly fit in and that’s okay. And then I could kind of see ways of working with it as a strength rather than letting it hold me back. [Autism is] not something you get rid of, while an eating disorder, people gave me the impression that if I recover from an eating disorder, my life would be, you know, the difficulties would all go away. I think my parents were quite, especially my mom, a bit guilty….just because, maybe because, she didn’t push even more to investigate I guess. You know, I don’t feel I can objectively say whether or not I am autistic, I really don’t, because I’ve never really understood what it should look like. I didn’t really feel it was me, but then again I didn’t really feel I had enough information to make an informed judgement…. when it’s been undiagnosed for decades and I’ve had to function, which I have. With this information I was then able to explore all the difficulties. I had the words to communicate, whereas before my way of communication was very much by not eating. That was kind of a bit of a turning point in that [therapist] understood like how best to communicate with me so she gave me written summaries after my appointments, she gave me written information, we used kind of goal setting to help plan my care. So [the inpatient unit] created a different space that was separate, because I think they realised for my stay there, it wasn’t appropriate for me to go through the whole treatment programme. ‘I can be a bit more compassionate to myself because it’s not my fault that I find certain things difficult’. ‘It wasn’t closure, it was almost like that was the start for the next chapter of my life’.
(Downs, J., & Adams, M. (30)). Re-imagining connection: the role of late autism diagnosis in eating disorder recovery and social support. *Journal of Eating Disorders*, *13*(1), 120.	Narrative and autoethnographic methods	The social dimensions of illness when undiagnosed Treatment experiences when undiagnosed The process of diagnosis The role of social support in autistic ED recovery	I was overcome by the bright LED lights and the basketball court’s reflection. There were at least 100 students in the gymnasium, most older than me. I was equally overwhelmed by how the laughter and loud voices echoed around me. I turned my increasing anxiety and feelings of isolation inward, making athletics and exercise my passion, the “special interest” I was focussed on becoming the “best” at. I began to think of my eating disorder as a friend. Something that was always there, something predictable that gave me a sense of identity. A big part of my experience of the social world was shaped by a sense of ‘not knowing’. Not knowing why I found it so difficult to do things that other people seemingly were able to join in with. Not knowing how to cope with constantly feeling overstimulated on a sensory level, whilst at the same time being under-stimulated intellectually. Not having others know how and why I was struggling so much. This got me into some very difficult situations where I didn’t know what healthy boundaries were, and was at risk of being exploited. felt like spending my life on the outside looking in at the kind of life that I wanted to have. I found a very effective way of being able to cope with my distress in social environments was to dial down the intensity with which I was feeling everything. That meant numbing my senses and suppressing the vehicle for feeling - my body. That’s where my eating difficulties really began. It took days to adjust to the unit schedule, rules, other patients, and the physical structure of the unit. Paradoxically, I later found solace in the schedule and rules. The predictable way of existing that treatment provided was upended when I was discharged. The drastic change in environment, support, and routine caused severe anxiety and a sense of chaos. I soon returned to my eating disorder ‘friend’ and the predictability it provided. I can see how my eating difficulties arose within a context where my differences - whether in sensory experiences or communication styles - led to exclusion from social support, a lack of belonging, and marginalisation from understanding, both from others and myself. Instead of helping me untangle these challenges, treatment only reinforced my sense of invalidity by failing to provide the insights I now have about my experiences and needs as an autistic individual with co-occurring neurodevelopmental, psychiatric, and physical conditions. In treatment, I went from not knowing why I was struggling so much to being given explanations that didn’t fit - explanations that seemed more like stereotypes than reflections of my reality. By the time I reached treatment, my ability to mask my differences was so ingrained that even the experts couldn’t perceive them. It took many years before I received a diagnosis and, with it, a doorway to self understanding and a better ability to meet my needs in recovery. I pursued an autism assessment because my major depressive disorder and ED did not seem to account for all of the challenges I faced. it was enlightening to reflect on how connected autism was to my ED. I think the onset of my AN was, in part, a strategy to deal with the overwhelming transition from childhood to adolescence. AN became part of me, something I was interested in and had to ‘perfect’. This period of discovery felt like a puzzle finally coming together, providing clarity about struggles that had long felt inexplicable. At the same time, it was a destabilising process that challenged my self-concept. It felt almost like grieving – not only for the parts of myself that had been misunderstood or overlooked by others but also for the opportunities I had missed because I hadn’t fully understood or accommodated my needs. The diagnosis gave me a language to describe and communicate my experiences in ways I hadn’t been able to before. It allowed me to re-story my life, reframing my differences as expressions of neurodivergence rather than failures. Forming relationships with people I’ve initially met through online platforms has been valuable. I’ve connected with friends and colleagues with similar passions, whereas navigating that for the first time in person can feel impossible or irrelevant. Another integral part of support has been my close relationships with animals. My dog helped me through the worst of my mental health. At times, I became emotionally exhausted, and I would lie in bed for hours. My dog, who I loved so much, sensed I was hurting and refused to leave my side. My experience of social support might be different, not disordered. Understanding I am autistic has enabled me to work with my differences, seeing them not as weaknesses but as variations that could exist alongside meaningful participation in social activities and relationships. I no longer feel compelled to be the same as everyone else or to mask constantly in an effort to fit in. Instead, I can see how I have performed inauthentically in relationships and social situations in ways which often haven’t been supportive of my recovery. Being autistic doesn’t mean I should opt-out of social engagement or don’t need to find a sense of community and belonging. I have been able to learn from peers who also navigate multiple struggles including with their health and social experiences. Ultimately, being able to find belonging, experience reciprocal caring relationships, and feel valued and understood for who I am has been a profoundly reparative and healing process which has challenged the deep-set ideas about myself that entrapped me in my ED in the first place.
(Healy, S., Pacanowski, C., Kennedy, L., & Obrusnikova, I. (31)). “This cage that I’m stuck inside”: Autistic adults’ perceptions of weight management, body weight, and body image. *Autism*, *25*(7), 1985-1998.	Semi structured interviews (dentified via Interpretive Phenomenological Analysis	Mental and physical health: direct and indirect interactions ASD traits and weight management Body dissatisfaction: unrealistic ideals and misperceptions Sources of information	my job is also very stressful so when I get home, I don’t want to do anything. I just want to sit in one spot and not move if I don’t have to”. And yeah, I just find comfort in food. So, I just eat fairly constantly and not very healthily. I think have somewhat intentionally dissociated my personal value from how I look just, so I don’t have to feel bad about myself. So, honestly, it [being overweight] means a lot. It means a lot for self-esteem. “… I wanna look not like I have a beer belly. But it’s not just the looks. It’s more that I wanna be healthy, and actually enjoy life, and be able to go do the things I wanna do”. “Well, I’m interested in the way I look primarily for how it impacts my mental and physical health”. And then, a lot of us [autistic adults] struggle with certain things like depression, and depression can really affect eating habits and activity habits. After I got laid off, the depression I was feeling really started to have an impact on what I was eating, and when I was eating, and how much I was eating, and I gained probably eight or nine pounds in a fairly short period of time. I look at myself and I’m like, wow, I got fat, and I feel gross, and I don’t feel good about myself. And I guess I go into that whole valley of eating my feelings kind of situation. “I can easily spend hours playing video games, working on genealogical research, reading, or just daydreaming … My interests can easily become obsessive”. “I’m obsessive about the numbers. I weigh myself every day. I was so obsessed with counting calories and eating as little as possible because I was so fascinated by the dwindling numbers on my scale that I ended up losing 40 lbs in 2 months. “I guess I just get really anxious because I don’t know what I’m doing [when exercising]”. “We [autistic adults] don’t like being around crowds, or going to the gym is not something that really appeals to us. In fact, it is quite horrifying”. “At these events, there’s food. So, that’s something I sort of go to … so I can just do something with my body that’s not talking”. People just stress me out and exhaust me, especially in real life, less so online. So, it’s difficult for me to reach out to other people for support and commiseration, which means I don’t really feel connected to other people when I’m trying to lose weight. It can be difficult to eat a balanced diet because green leafy vegetables freak you out. “I’m a creature of habit and I get into patterns with food that are generally bad patterns”. “I can only eat very specific foods at very specific times”. The best way to explain it is I just kind of have this clock or schedule of the way that my life works. Food is one of the most important things. A lot of the reason that I don’t travel is because I can’t control the food that I eat. I won’t go on dates if I can’t eat the food. Every Wednesday I have to eat Chinese food. I think it’s my sedentary lifestyle, and also I eat a lot of junk food. And in general, I guess, it’s those things, but also a lot of other things, like genetics, and certain medications I know can cause you to gain or lose weight. And certain medical problems. “I’m totally not in love with it [her body]. There’s so much work which has to be done that I just wish that I could zap myself into oblivion and start all over again with a brand-new body”. “I would like to be less, I don’t know, potato-shaped”. I hate my skin so much that I want this ink [referring to her tattoos] inside me and I want it to like [inaudible] and I don’t want it to look normal … It [her skin] mostly just feels like a cage to me. And so, it [tattooing her body] is just a way for me to decorate this cage that I’m stuck inside. I have a hard time remembering what I look like … If I try to conjure up a mental image of what I look like, and then I go and look in the mirror, it always surprises me a little … It’s always I look bigger than I remember. “If my family or friends think I look healthy. I’m allowed to feel good about myself”: Brad. “But sometimes if I’m trying to look nice, then I worry about if other people think I look nice”. Everywhere I go, I’m constantly bombarded with messages about how women should be slim and pretty, otherwise they’re just hideous sacks of flesh who have no value or purpose … So obviously that sort of toxic ideology does have an impact on how I feel about my weight. like, oh, if I looked good, at least I would have that because I don’t have anything going for me socially … I wish I could just be a pretty face because I feel like there’s not much else that people see in me. As I started to lose weight, I really noticed that people make eye contact with me more. People would like start holding doors for me. People are just in general nicer to you. I don’t really understand why but –I’m kind of used to not fitting in based on other reasons, but once I lost weight, that was one less reason. I’m finding out information on YouTube and Reddit and also Facebook support groups. I do independent research too”. “I’d done my research the second I found out I had diabetes. I spent the better part of three days researching information on every aspect of it”. “Thinking that the doctor, usually you just get the generic, “Oh, well, you know, it’s all you gotta do is exercise, eat less calories than you consume, and drink more water”. “was telling me about how I would lose weight by going on this diet. I was like, “Well, that’s not actually what I’m here for,”. I have a medical doctor, a counselor, and a psychiatrist. All three work with me on my diet and my sleep and exercise”. “… the advice that they give me is very individualized to me”: “how they [medical professionals] interact with you in the space that you’re in is really important…. I’ll try it, and then I’ll burn myself out, and then I’ll be off it forever”.
(Kinnaird, E., Norton, C., Pimblett, C., Stewart, C., & Tchanturia, K. (14)). Eating as an autistic adult: An exploratory qualitative study. *PLoS one*, *14*(8), e0221937.	Interviews Identified via thematic analysis	Autism and eating Impact	“if the texture is off, that food can be just fantastic and I’ll still loathe it”. “I’d rather not eat in a loud restaurant or dining hall, or just anywhere where I can’t talk to people next to me or just have some peace and quiet. “I don’t like the smell sometimes that forks and knives have on them. So I will insist on clean cutlery, and if it smells peculiar I will send it back and get clean ones. And in fact sometimes mugs out of the dishwasher have that smell in them as well, and I don’t know what that smell is but it’s pungent. And so if it doesn’t smell just right I won’t eat out of it or on it”. “it’s the immediate feeling of being full. You know, two bites and you’re full, that’s it. Even if you were a bit hungry, two bites and you’re done”. “Planning and doing things in the right order can be a big barrier to accessing food. For example, I don’t leave my room unless I have a visual mental plan of exactly what I’m going to get, where it is, and how I’m going to get it (often with a backup plan or two so that I don’t panic if my first plan is disturbed), and if I don’t come up with a satisfactory plan I just don’t go at all. once I start executing a plan I enter a sort of autopilot mode, and the system gets lost if it’s bumped off course. Oftentimes when that happens I quickly give up and pick something at random to get out as quickly as possible, and that can result in accidentally picking something sensory-bad”. “literally forgetting to eat because I just get too busy to, I don’t know, recognise the signals”. “you’re quite rigid, you’re quite kind of control orientated, you’re quite kind of perfectionist”. “I think I’m in control of everything simply because I’m in control of the facts, and I know exactly why I’m doing what I’m doing”. “The milk was sour at school once, and I wouldn’t drink milk for a long time after”. “[It is] easier if I don’t have to think about something new to eat. If I know something is good, and that, you know, I’ll feel ok after eating it, then I’m likely to eat it again”. But I don’t want to eat like that so you know, I don’t feel inspired. I feel a bit repulsed actually, just sit back and observe in grotesque curiosity I suppose”. I always prefer to eat alone rather than with people. Eating is not about “hanging out”, it’s about “putting food in my belly so it stops complaining”. Because I started eating it at a certain time of day and then I kept doing that for a long time and I was gaining weight and I noticed that I was constipated and I had to stop that habit. But it’s very easy for me to get into a habit”. “If you’re not taught how to eat well, and you know the sort of behaviours you see as a child is emotional comfort eating, I guarantee you that’s what my mum does. Then that’s the sort of thing you’re exposed to, you know, having a bowl of cereal sized for six people before you go to bed sort of thing, or you know, asking for a bowl of ice cream and you get the entire tub before you go to bed, just because somebody’s upset you you eat a candy bar. You know, that sort of thing, if that’s what you’ve been exposed to, which I was, then that, the sort of patterns that you assume”. “We’re all so literal, and all these messages that are out there and all these skinny girls on Instagram- now it’s the bodybuilders on Instagram, you know, the fitness freaks on Instagram … I think these things are all around us, I think we’ve got to think about them very clearly and carefully. Particularly when we’re thinking about how literal autistic people take these messages”. “Autistic people often misuse “black and white thinking”, and I know that affects what and when I eat at home, because if I go to get a snack and my mom says it’s too sugary or it’s saved for something/someone else, I don’t get a different snack, I just don’t eat. I think that sort of thing contributed to my eating disorder at the start”. “I definitely have a tendency towards completion that will cause me to finish a meal that I am not necessarily hungry to finish, and that may be one thing about it. There’s definitely a compulsive element to my eating a lot of times. I also think that, you know, there’s an impulsive element to my eating, and they both sort of come together to, you know, create binge episodes when they do occur”. “For a long time I would eat only foods I recognized. I worked pretty hard to overcome that … and I have learned to like the experience of trying a new thing, even if it may not be what I know … they’ve improved because I worked at them”. So I feel like that habit helped me diversify my tastes and it definitely gave me courage to try new things”. “I think not knowing that my body weight was actually linked to how much I put down my neck- no one ever told me that the two were linked together- was just a big issue as well. So I think this has been a lifelong thing for me. Never knowing that the two were related, never knowing it was something that I had at least some degree of control, responsibility for. As a responsible adult. I think once I discovered that about five or six years ago and once I discovered that it was something that I could change and I did have some power over, then that was empowering in itself and that’s when I started to take some control over it”. “making meal plans helps, I think, because I then kind of have to—I don’t get into a panic about “oh, what should I eat”. I’ve kind of got it all sorted out before”. “They also know about my “nope” reflex. Some things just make me go “nope” and they don’t chase me with them or ask me to try them anyway … We all abide by these boundaries and it helps tremendously”. “My experiences, be they atypical, are still perceived as valid”. “I think it would be helpful if more professionals were aware of the sensory differences and the frequently-comorbid gastrointestinal issues that can make eating difficult for people with autism, as well as the samefooding phenomenon and other routines. Also, in general, they need to understand that meltdowns and sensory overload aren’t tantrums or bad behaviour and can’t be helped by treating them as such—especially with food, it’s not as simple as being picky, it can range from unpleasant to uncomfortable to painful”. To know that there is a category of people who have these eating issues and they don’t actually have anything to do with the shape of their body. And to tell them that they need to change is wrong. It’s ethically wrong and it’s going to ethically fail, it’s going to cause these people more damage. It’s not the best way to go about things”.
(Kinnaird, E., Norton, C., Stewart, C., & Tchanturia, K. (32)). Same behaviours, different reasons: what do patients with co-occurring anorexia and autism want from treatment?. *International Review of Psychiatry*, *31*(4), 308-317.	Interviews Identified via thematic analysis	Relationship between autism and anorexia Problems with treatment Treatment adaptations	I’ve never really had the thoughts of I want to, kind of, I don’t know, be really thin or lose weight. I’ve never really—like it’s always just kind of happened. it is a bit about body image that everything like that but … It’s more kind of a way of dealing with sensory things like, yeah, it’s a massive thing of not knowing that I had sensory issues and it as a way of coping with those. The first facility I went to, before the autism diagnosis was problematic because they thought that I was stubborn and lazy and unwilling to help myself, and they let me know it. They ended up asking me not to come back, because my case was too “complex”. I’m very very sensitive to noise. You know, extremely sensitive. Like things like laughter and stuff, I can pick it up so easily and I don’t like loud noises, like when someone’s distressed in the room I get very distressed, it makes me feel distressed…. I feel like they didn’t understand that I needed to walk out, because apparently, they handed it over to the other staff for my notes. I feel like they don’t understand I took myself out of the situation because I can’t cope with the noise. It was sensory and texture and just, caused me so much anxiety not because of what food it was but because of like the sensory aspect of that food … it was always kind of acted as though I was just not really aware and it was really the food that was causing me distress. And then that made me more angry because it felt like they were saying I was lying. Treating the AN as though it exists in a vacuum is incredibly harmful, because it’s demoralizing to the patient, it delays recovery, and it frustrates everyone involved. Emphasis needs to be placed on figuring out which behaviours are anorexia based, and which are autism based. If someone is refusing to eat their dinner, it could be because their eating disorder is telling them that it will make them fat, or, the food could be touching, is an autistic sensory issue. The behaviours are exactly the same, but the causes can be so different. Knowing all of this, if doctors and therapists and dieticians can be flexible regarding autistic patients, they’re going to see much better outcomes. this is why, this is why I’ve felt like I’ve not fitted into the world for the last 45 years, and actually I’m just different and that’s ok. I have like sensory issues with touch, for example textures. But that’s something, like last time because I think I was masking I wouldn’t bring it up. And it takes quite a lot of courage to be honest and say “actually, yeah, that’s a sensory thing for me”, like last time I kind of thought “oh well to recover you have to mix your foods” so, I don’t know, it was almost like masking the recovery. I know that their model of therapy is not going to deal with that. I, so, it’s not going to address what I think. I think as somebody who’s on the autistic spectrum and who has an ED, I think AN is kind of like a product of who I am and how I think. but people getting that like someone is going to still have, probably, a rigidity around their food, like and that not being totally—not just in inpatient, in outpatient as well—that’s not the battle to be fighting. It should be about how much is that person able to now be like engaging in life.
(Li, Z., Chubinidze, D., Croft, P., Webb, J., Sarpong, A., Zesch, E., & Tchanturia, K. (33)). “Don’t talk to me like I am an illness”: exploring patients’ needs using the communication passport in an eating disorder service. *neuropsychiatrie*, *39*(1), 28-35.	An explorative qualitative analysis of 38 completed communication passports	Communication preferences Sensory sensitivities Special interests and strengths Important message to the care team Dislikes and struggles Support needed	“calmly, kindly, gently” with a “soft voice”. “Sensory needs (e.g. my sensitivity to light, sound, touch, texture, taste, or smell and how you can support me)”, “slamming doors”, “alarms”, “footsteps”, and “loud music on the radio” stressful, “Low light helps me focus. Bright lights are distracting”. “need flavour and need sweet”). My family and friends all mean a lot to me”. “Treat me like a normal person”; “Don’t talk to me like I am an illness”. “I want to get better”. “[Dislike] schedule that is uncertain”; “[Struggle with] processing information”. “I struggle to ask for help”. I don’t like being forced”: “[Dislike] being patronised”. “[Struggle with] giving myself permission for enjoyable activities”. My dislikes and things that I struggle with, and how you can support me”. “The reassurance that it is okay to eat my meals”. “If a plan is going to change, please tell me”. “Check in with me”; “Asking me what could help in the moment”. “Help by talking about other things outside of [ED]”. “I need time on my own”. “Keep me in the loop”; “Be collaborative with me”.
(Makin, L., Meyer, A., Mondelli, V., & Tchanturia, K. (34)). Regulating with food: a qualitative study of Neurodivergent experiences in adults with binge eating disorder. *Journal of Eating Disorders*, *14*(1), 13.	Interviews Identified via reflexive thematic analysis	Understanding the Neurodivergent self irregular eating leading to bingeing binging to self-regulate personalised and adaptable support	“I don’t know” [026] and “I haven’t thought about it” [022], and wished clinicians had explored this with them, saying “because it’s something that hasn’t really been discussed or looked at, it’s not something that I’ll know what needs to be managed”. “there’s no way to, like, say, oh, it’s this or that. So, for now, it’s just, like, damn”. “It didn’t come up in therapy because I hadn’t thought about it. But if they were to [ … ] bring it up, I think it could have made things a lot more clearer for me. wow, like, there’s a lot of things that could be like, yeah, parallels. And there’s a lot of explanations that were applicable”. “once you understand the reasons that you do stuff, then it’s much, much easier to change your behaviour”. I might skip lunch there because I didn’t like what was to offer and there was no alternative that I liked” [009] and “I was a finicky eater and then I’d kind of eat once I discovered what I felt I could eat”. Gets like a lot of consistency. But I know it takes exactly 3 hours. And so if I don’t have orange juice ready, I will wait until I have that orange juice. I’ll wait for it to slushify before I eat”. “I think that when I’m hyper-focusing I forget everything else, so I don’t eat or take care of myself. And so, I mess up my eating routine and then I’ll binge to “make up” for what I didn’t eat”. “I think something that led to bingeing is also not recognising that I’m full. [ … ] I think my whole sense of like interoception is not great”. “[bingeing] was probably like sensory seeking behaviour” [002], with eating having “become a bit of a sensory thing”. “wholemeal bread and butter, like slice after slice, because I really liked the contrast of like the texture”. “swallowing specifically or like just having a feeling in my mouth” [032], or the “sensation of chewing”. “recognising that I just want the sensory input and I’m not actually hungry, helped me to choose more vegetables”. Maybe I need a little shot of dopamine before I do the task [ … ] So that might be like crisps or drinks and really icy cold, sparkly water. and don’t have to worry about the people around me and have my food in peace”. I just do it, I don’t think, I just do it and then when I’m doing it, that’s when I think ‘Oh, ****’ [ … ] And I’ve been known to travel far and wide to find a specific cake that I want. “Neurodivergent people have different needs [that] might change over time”. “I do think CBT can be super helpful for some Neurodivergent people because it’s very structured”. “I think possibly having a community to turn to of people going through similar struggles would be nice”.
(McIntosh, A., & Hunter, R. (35)). Exploring recovery from anorexia in autistic adults: a qualitative study. *BMJ open*, *16*(1), e111034.	Semi Structured interviews Identified via reflexive thematic analysis	Recovery in progress Changing to healthy mindsets Sensory experiences Engaging with treatment	“not a linear thing” (P5) and they have “had slip- ups for sure”. I guess that is just part of recovery. I choose to eat and do the right thing even though my mind would still prefer to weigh 80 pounds. I struggle with that word ‘fully’ because I do think I am recovered … there’s this kind of ‘its definite’ and you’d never be vulnerable to that illness ever again which I think that’s kind of naïve thinking … like any mental health problem or like addiction it’s like an automatic thing to go to. However, I do still have parts of me that are anorexic and still in anorexic thinking. It was a lot due to my parents commenting on my weight … it wasn’t just my parents, like there would be relatives stating if I gained weight, or if I lost weight and they made me feel conscious. [If] I see that they’re eating, I’m always tempted to consciously eat less than them. looking at content creators who focused more on body neutrality and body positivity, rather than looking at influencers who’s like ‘you can lose this much weight in 2 weeks. I am extremely privileged, and I have two sisters and several close friends who know I am … bonkers … and still show up. taking lower stakes work and managing how much work I have and how much I’m juggling is important. makes you convince yourself that you’re not worthy of anything or not deserving of anything”. It kind of seeps its way through to different sort of elements of your life. [It] affected my self- esteem, and I would tell myself how stupid I was or how I did not deserve to live, or how I did not deserve to eat. So, I would not eat. “it’s very hard to break out of those … thoughts that led to the eating disorder … very hard to break rules”. I often have nightmares too. Not only about weight, but about not being enough or the consequences of eating or people like dying and things … once I dreamt I died, and everyone was talking about how fat I looked at my funeral. “don’t have to suffer anymore”. I’ve essentially had to rewire my thinking … I am not losing control by following a meal plan … I am making the conscious decision to eat … I am in control by saying that I will do these things. I learnt to not be so hard on myself …. Weight doesn’t give you the full picture, it doesn’t give you your body fat percentage, it doesn’t give you like the strength that you have from certain physical activity. I try and actively avoid triggering content on social media, news articles, thinspiration stuff. Social media and TV shows and movies … blatant fat phobia. And the way society makes it very hard to be okay with being in a larger body … there is no size that is healthier or attractive than another. And people in bigger bodies can be just as healthy, or healthier than people in smaller bodies, and just as beautiful. I don’t think I’d be able to stay in recovery if I didn’t have my support system. My parents have been so supportive of me and my struggles with mental health as a whole in the past, I almost feel like I owe it to them to at least try … I missed out on so much when I was anorexic. … just being with someone while eating, and where eating is something pleasurable, has been very invaluable. We are very far apart but we video chat with each other sometimes and eat dinner together, The way that my body works in terms of sensory … at all times I feel every single piece of clothing on my body, no part of it ever goes away. I didn’t have a normal relationship with food … I don’t communicate very well with my body, so I don’t feel hungry necessarily and I don’t necessarily notice or make the connection that I’m being hypoglycaemic because I haven’t eaten. I knew I was upset about gaining weight, but I wasn’t upset necessarily, because I wanted to gain weight … It was just very confusing. Everything just felt wrong and that led to a lot of anxiety around my body and around existing in public spaces and a lot of depression from that disconnect from my brain, from what I was and what I should be. It’s also not disordered behaviour, it has nothing to do with me choosing to eat or not, it’s just a fact of life. It’s how I react to this experience. One participant described the distressing experience of smelling a food they avoided as being like an “extremely high- pitched squeal at all times”. It is frustrating because even when there are times that I want to eat or foods … if my senses can’t handle it, then I just can’t do it. I feel a lot more comfortable if I can just taste every component, then the whole thing all together. “gives a sense of control, a sense of ownership, a sense of predictability”. I really do like is the routine. I do like knowing exactly what I’m going to eat, with who I’m going to eat with. being autistic definitely made the depths of my obsession worse and the obsession was the main root of the anorexia. I’m also very rigid and categorical in terms of needing to have earned eating food. Who knows, without the autism, and by connection the obsessive nature that comes with it, maybe I wouldn’t have developed anorexia at all (. It scares me very much when I’m not able to access myself, like my brain, like I’m used to doing. I felt like I was dying … My body was shutting down, and my doctor said I had just a few more weeks to live if I did not get help. My heartbeat was slow, I had zero energy, I could not think. Nobody really acknowledged my autism for a few months … and they were like ‘nah that’s just your eating disorder’ … I wasn’t given explanations for things which made me really distressed and just having panic attacks when it wasn’t about the food, it was about … nobody giving me clarity. “you don’t look anorexic”. “there were parts of it that didn’t really land with me”. but it didn’t really help me deal with the sensory difficulties of … having my body changing rapidly or, you know, eating a lot of food, or dealing with digestion. They were very focused on whether or not I thought I was overweight, which I’m not delusional I know that I’m not, and I never had a specific like desire to look a certain way … I also think it’s pretty harmful to have that as such a big criteria. “containment and the structure of the [treatment] ward was definitely helpful and not being solely responsible for my own care”. “steps to follow and order”. a test for me to see like if I could be responsible for myself and it gave me that chance to really like prove it to myself.
(Nimbley, E., Buchan, K., Maloney, E., Kettley, S., Sader, M., Duffy, F., & Gillespie‐Smith, K. (36)). Using Photovoice methods to set research priorities with autistic people with experience of an eating disorder. *Journal of Clinical Psychology*, *81*(8), 677-693.	Research questions via photovoice Identified via thematic analysis	Impact of early experiences Function of the ED Barriers and facilitators to ED recovery Understanding and accommodating for complexity Changing research culture	“what happens when we are younger fixes so much as we [get] older”. I was taught that it was important … you know those messages would affect anybody. But it’s like as an Autistic person who is learning to navigate the world through figuring out the rules. A really powerful image and it sounds like it resonates with a lot of us. The rules we tend to make or create in our minds and they become so like powerful, they’re so important to us. It’s like hard to justify like why I should have this type of food or this amount of food or this kind of brand of food. And then, I don’t know, I just feel like the rules cannot be broken … it’s that kind of black and white thinking, like, no, you cannot break it. Whereas I’ve heard experiences from those who have eating disorders not coming from that autistic kind of lived experiences. It’s not as, as much, if that makes sense. How do we break the generational cycle of eating disorders? How do we sort of make that support so that the younger people that are coming up through life now are able to kind of realise, like, how can they have sort of a healthy relationship with food, what can we do to kind of stop that continual thing? I guess for me that picture is about trying to break that cycle. It’s about trying to create something for my children that is different. So, I guess that picture is one moment where I feel like I managed to do that. You know, one moment where I managed to put my own shit behind me. I was trying to get across that food fuels my brain, [and] sometimes I don’t want to think so fast, so I’ll reduce the amount I’m eating to slow down my brain a bit. Interesting for future research that three people with autism find that they reduce [for] their brain activity. That was what my psychiatrist think is the whole cause of my ARFID, reducing food to cope. It was something that I sought comfort in … I was definitely seeking comfort and escapism from those kind of overwhelming feelings. I think that’s probably the best way to put it. “to a point where my body wasn’t doing anything that wasn’t absolutely necessary to keep me alive and emotions were something that I almost stopped experiencing”. “problems with processing and expressing emotions, or alexithymia. I really idolized her in a way … And it was like, yeah, I think that added to it in itself of almost like, you know, trying to almost mimic that. Being like, if I was like her, I would fit in. If I was like this other popular person who also looks like this, I would fit in. [Being thin] just felt like it was taught everywhere, and it was a way of fitting in and being accepted. And at the same time are trying to fit in and, and trying to sort of meet an ideal of what they think that, you know, you’re trying to act like you think other people think should act, in the same time, you think ‘I should look like a normal person’, you know, especially when there’s, you know, you feel the societal expectations on how you should look as well. [Sensory experience of clothes] makes being happy about all the things I can do difficult because I can’t go out the house because I feel so uncomfortable in what I’m wearing. So then that freedom I’m supposed to have now isn’t really there. And I think, you know, finding a way to help with that side of things and making that process easier would, would be really beneficial. “is a whole other variable to a situation that I did not have to think about before (and it turns out I get very overwhelmed very fast when I’m too hot)”. It feels like textures on the inside and I can’t get away from that. And like that’s, that’s the problem. I’m Autistic, I’m going to approach my recovery like I’m Autistic. I feel like being Autistic [can] sometimes be a blessing and a curse in terms of recovery and relapse and both whatever. But, like, that actually being routine really almost helped? I felt like if I’d had that support and continue with, to maintain that routine and that recovery … that like genuinely I would probably mostly nearly be recovered from my eating disorder. I found routine and reminders extremely helpful to avoid eating too much throughout the day. Becoming more flexible in my thinking this was not something I was going to be able to do as I naturally have a preference for routine and familiarity. Attempting to change this or challenge it in some way would have created even more distress to me and wouldn’t be something I would be able to maintain. It was important for me to understand the difference between what anorexia was controlling and what was just my way of being. “personally for me, it has actually been useful to try and separate those aspects of my life and impact that they have on my eating”. Just to kind of reflect on the kind of figuring out thing like … I sometimes get really frustrated with people like now when people are kind of like ‘oh is that your autism or?’ and I’m like ‘I don’t freaking know’ like I’ve only been me … I don’t know how there was something that I can separate out and there’s some things not and it’s sometimes really frustrating. you can’t really separate the two…100% we have to stop trying to separate and just have everything together and look at it as the whole person. I don’t know why but it makes me so angry”. if it’s useful for you to pick it apart, if it’s useful for us to pick it apart, then having professional support to do that is really, really useful. But if someone else is trying to pick it apart to minimize something or diminish something or challenge us in a way that we don’t find supportive or helpful, then maybe that’s not a helpful route to go down. To treat someone, you do need to look at cause and I think if you took me and someone else with ARFID, then our treatments will be so incredibly different because of the different routes and you know, there are so many different reasons for I think like categorising people, not just by their symptoms but by, how this has come about it’s going to be a lot more effective to treat them. consistently missed both by GPs, by eating professionals by every other professional … and that is simply because they weren’t aware. It’s really common [in Autistic people] to have like digestive related problems … and then also if you have an eating disorder and then you try and eat in like quote un‐quote ‘typical ways’. you quite often have like all sorts of digestive chaos as well … It’s like, so I said in hospital, like, ‘I feel better when I don’t eat.’ And they thought I meant like somehow like mentally or something, I feel better. And they were just told me that I didn’t. And that was just completely dismissed. think that a definite research priority that needs looked into is why are those co occurrences more likely in Autistic people and how does that link in with treating the whole person? I think that’s often overlooked. I think if they have the right education and understanding of what [co‐occurrences mean] and why they might be, it can just hopefully start those conversations and improve that awareness, which is really important. Especially in my culture with within the‐ Indians and South Asians, there is that kind of culture of when you go to families and parents that love eating or overfeeding someone. And it’s I guess their kind of hospitality or they just they want to kind of welcome and have, have people around and I suppose [they] use that food as a social aspect of gathering. I just want to agree with that as well. I think that like, like you say, the sort of like different cultures aren’t really like spoken about or acknowledged in like treatment and like teams. I don’t think anyone told the nutritionist that I wasn’t a girl. And they basically saw me and went I don’t really know anything about male biology and then this is kind of specialised for girls, sorry. I think we over gender it sometimes. We talk about male eating disorders, and I’ve never seen someone talk about male eating disorders that isn’t to do with gyms or body image of muscle dysphoria … and it’s just not true. We try to categorise things so much we lose sight of the person, and I think it’s just the person we need to be looking at. I’m hoping that, like, the training and priorities of treating people with autism and eating disorders is welcoming everyone’s point of view and it’s not like discriminating against anyone … I guess what I’m saying is that I think that it’s really important to not judge anyone and not discriminate against anyone and welcome everyone with more open arms because from my experience sometimes that’s not happened. I think if practitioners in this area are more informed and or more individuals are upskilled to work with people with autism and associated eating disorders that this could potentially be life changing for some. I also think that as part of developing any education for professionals, it would be important for them to engage with those who’ve got lived experience. I think for me, real recovery happens in the real world, and I think a way to prevent that is really where the research should be, or at least making community support robust and looking at ways for it to be adapted for people with ASD. I think a research priority is just trying to get the right support in the community for people and how to make that better, how to keep people at home, not let is escalate.
(Nimbley, E., Gillespie-Smith, K., Duffy, F., Maloney, E., Ballantyne, C., & Sharpe, H. (37)). “It’s not about wanting to be thin or look small, it’s about the way it feels”: an IPA analysis of social and sensory differences in autistic and non-autistic individuals with anorexia and their parents. *Journal of Eating Disorders*, *11*(1), 89.	Interviews Identified via Interpretive Phenomenological Analysis	The Social Self Feeling the unknown Sensory foundations	I can only realize now but how much I struggled with social interaction and kind of things like that. I’ve always felt really really different, and I didn’t really know why…. just the way that I feel about myself, I think that’s a big part in feeling … um … I guess not feeling like I deserve or need to eat. There’s some distortion there like is occurring on my side. It’s not my ability to perceive those emotion but it’s just that distortion about myself and the way people feel about me that kind of interrupts the processing of that. I think when my diagnosis it was kind of a weight off my shoulders. Like okay with the socializing example, like I knew blaming myself … like I was doing something wrong and I was finding this more difficult than a normal person so clearly there is something wrong with me. And when I got the diagnosis it was like okay, I’ve not done anything wrong it’s just my brain works differently and I can now put things in place, try and like help myself in a way. Actually, the first time I think since starting school I felt that I could relate to my peers was when there was a particular group of us in inpatient care … who I think were all wired the same way. Very uncomfortable. Not just like, I think with a lot of the sensory things it’s not just a case of a like or a dislike. I used to explain it as a full body response to it as opposed to oh I like that smell, I don’t like that smell. It’s because social interaction is an extra thing to have like to try and process that I’m already putting a lot of my effort into that that other things will slip through easier. Like the lights will affect me more or people talking in the background will affect me more and that kind of stuff. What I struggle with, and I struggle with it in my own life as much as anybody else’s, is actually knowing what to call this feeling and therefore what the solution to this feeling might be…. But I’m not very good at knowing how other, verbally, how other people are feeling. I just sort of feel like I absorb it like a sponge. And I sometimes feel in my own life,I often don’t know how to describe how I’m feeling, expressing my emotions is very very difficult. Um, and that makes … that usually means that I kind of end up bottling them up. And I guess I know when I’m feeling bad, or I’m struggling or a lot, but kind of really pinpointing what I’m feeling, I just don’t really seem to be able to do that very well….trying to express how I feel or my emotions, that is very very hard for me. If someone asked how are you feeling, I would know exactly how I’m feeling, but I’m not going to tell them how I’m feeling. I can tell you that I feel dizzy, that my stomach hurts, that I feel tired, that my mouth feels dry, but will not connect that to I feel hungry, or I feel thirsty. And I used, even when I was younger didn’t I, my eating would be on a routine so I knew that I had lunch so I would eat. It wasn’t like, oh I’m hungry. And if I’ve eaten something, it was usually for the sensory seeking side of it? So, I’ve had time when I’ve wanted something crunchy so I’ve had something crunchy, it’s not like oh I’m hungry I’ll have a snack. I’ve never really done hungry. I’m not very good at knowing when I’m hungry. It’s not necessarily that I don’t know when I’m hungry … it’s a bit hard sometimes to tell the difference? I know there’s a problem but I can’t tell you….I can’t tell you what the actual sensation is. Sometimes I can’t tell whether I’m really full or really hungry.
(Sedgewick, F., Leppanen, J., Austin, A., & Tchanturia, K. (38)). Different pathways, same goals: A large‐scale qualitative study of autistic and non‐autistic patient‐generated definitions of recovery from an eating disorder. *European Eating Disorders Review*, *30*(5), 580-591.	Online study with free response answers Identified via thematic analysis	Weight Restoration Lack of ED behaviours Thoughts and behaviours Cognitions Emotional responses Getting on with life Ongoing challenges.	“weight restored” “reaching a healthy weight”. “‘not binging/purging any more’, ‘not starving myself to match my mental im-age’ and ‘not restricting calories’. ‘not weighing myself. obsessively’ and ‘not tracking calories’”. ‘obsessive or ‘disordered ideas about food’, not interrupted with thoughts of calories and food’. no longer ‘constantly thinking about food and/or my weight’ and that ‘thoughts about food are no longer constant for me’. “‘no longer looking at “thinspo” ‘,’no longer trying to be underweight’ and ‘I no longer constantly want to be thinner’”. ‘recovery is healthy thinking patterns as well as healthy weight’. not occupied by ED thoughts and consequently not engaging in disordered behaviours’. no longer having a destructive relationship with food, exercise or my body. To no longer live a life preoccupied with my weight or my body and altering either through unhealthy means’. ‘[I’m] focussed on being happy [now]’, ‘feeling safe and comfortable’ and ‘I feel worthy of food’. Similarly, the concept of ‘learning to love yourself’ and being ‘relax[ed] around food and my body shape’. ‘I can eat without enormous amounts of anxiety and shame’, ‘I can eat without guilt’ and ‘[I] can eat on a regular basis without fear’. now ‘get scared about losing weight’, ‘food does not control me anymore’ and of ‘no longer being controlled by an ED’. being able to deal with symptoms’ or ‘my symptoms are now less intense … I know how to manage it’. And ‘to be able to binge once without causing a chain of negative behaviour’, I eat food now’ – ‘having a fully healthy eating schedule’, ‘I can enjoy unhealthy foods’, ‘eating whatever I want when I want to’ – ‘I do my best to eat 3x a day minimum’, ‘actively trying to keep a standard eating schedule’. healthy relationship with food’, ‘no longer choos[ing] food based exclusively on nutritional value’, and being a ‘healthy weight [and] able to eat freely’. ‘more like a remission in a chronic issue with the ever-possible potential of a flare up’. ‘I don’t believe I can be recovered anymore than a sober alcoholic can be not an alcoholic’. ‘still struggl[ing] sometimes’, ‘one is never ‘recovered’, but rather ‘in recovery’’. I still can’t eat many kinds of food’) and (‘binging less but not fully recovered’) retained ‘some disordered habits’ or ‘an unhealthy relationship with food’. ‘cravings and self-loathing’, (continuation of). ‘there are times that I can still feel it’s grip on me’. ‘actively living with disordered thoughts while being able to overcome those urges’. ‘probably substituted food for smoking’ instead of learning to ‘deal with emotions in more productive ways’. hyper-fixate on the healthiness of something’, and one gave a long list of her new rules for ‘acceptable’ eating: ‘following guidelines from fitness magazines, and now online blogs on vitamin sufficiency, if your body feels worse after eating than when you were hungry then don’t eat it.

- Author(s), year of publication.
- Data collection methods.
- Key findings and themes.
- Participant quotations.

### Quality appraisal

The methodological quality of included studies was assessed using the Critical Appraisal Skills Programme (CASP) UK (39) Qualitative Checklist. This tool evaluates aspects such as study design, recruitment strategy, data collection, reflexivity, ethical considerations, and analytical rigor. Studies were not excluded solely on the basis of quality; however, appraisal findings were used to inform the interpretation of results.

### Data synthesis

Data was synthesised using thematic synthesis (25). This process involved three stages:

1. Line-by-line coding of extracted qualitative data, including participant quotations and author interpretations.
2. Organisation of codes into descriptive themes.
3. Development of higher-order analytical themes that extend beyond the findings of individual studies.

This approach enabled the identification of patterns across studies while preserving the depth and complexity of lived experiences.

### Data analysis

The database searches identified a total of 424 records. Following preliminary screening and application of the review search parameters, 39 records were imported into EndNote for further assessment. Duplicate records were removed, resulting in 21 studies for full-text review. Full-text articles were assessed against the predefined inclusion and exclusion criteria. Eight studies were excluded at this stage due to employing mixed-methods or quantitative-only methodologies (n = 4), focusing exclusively on parent or professional perspectives rather than autistic individuals with lived experience (n = 3), or being review papers rather than primary research studies (n = 1). Following full-text screening, 13 studies met the eligibility criteria and were included in the thematic synthesis. The study selection process is presented in **Figure 1**.

**Figure 1:**
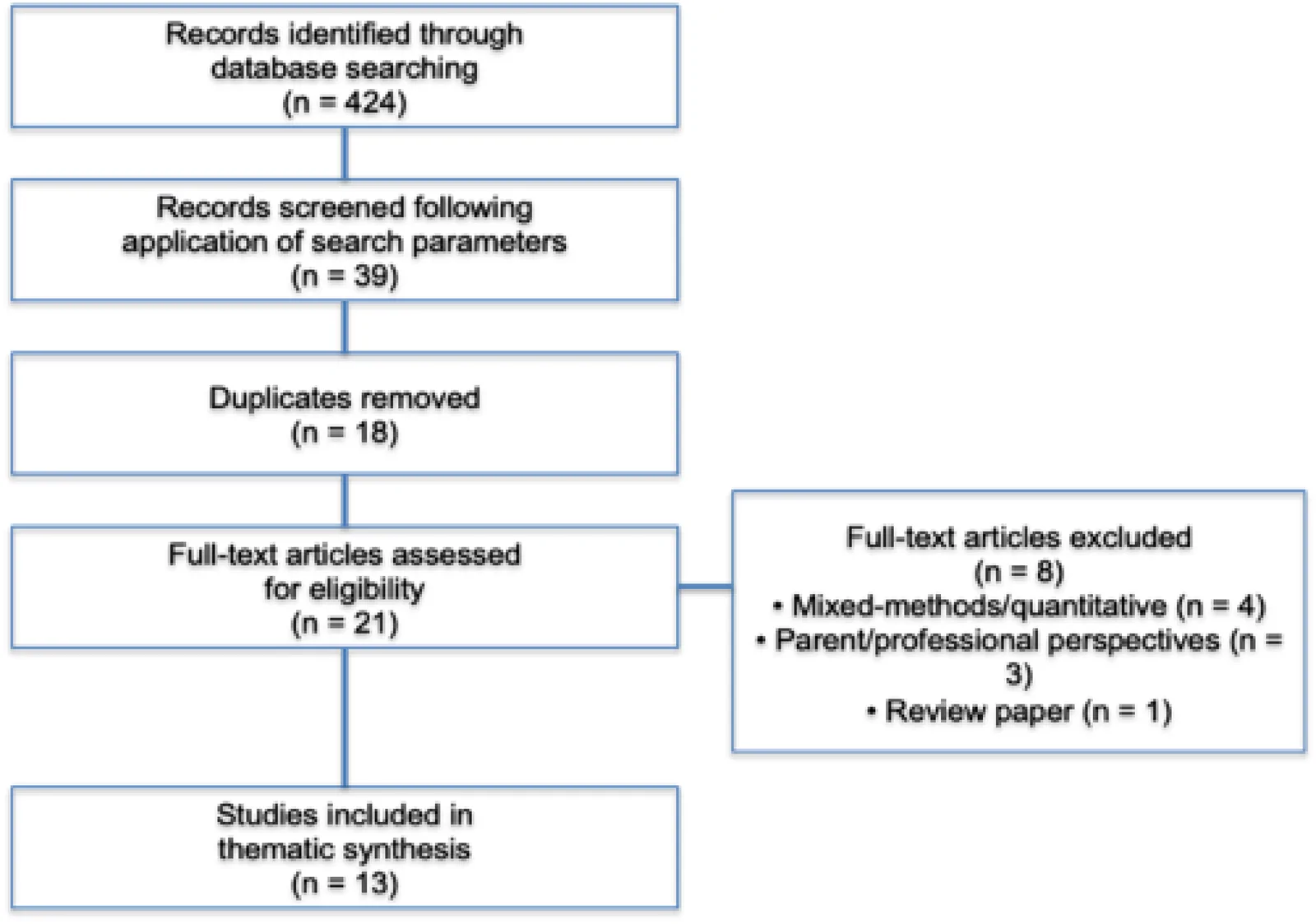
The Prisma diagram of the steps undertaken for this review.

Of the 13 included studies, six focused specifically on anorexia nervosa, four examined multiple eating disorders, one focused on binge eating disorder, and two studies explored disordered eating. This can be seen in **Table 3**. Two reviewers (BW & GB) independently appraised the methodological quality of included studies. Ratings on the CASP checklist were compared, and any discrepancies were resolved through discussion until consensus was reached. Initial agreement between reviewers was high, with disagreements identified on four appraisal items across two of the 13 included studies. All disagreements were resolved through discussion. A copy of the quality assessment rating table can be found in **Table 4**.

**Table 3:** Primary eating disorder focus of included studies.

Study	Primary eating disorder focus	Classification
(Kinnaird et al. (14)) *Same behaviours, different reasons*	Anorexia nervosa (AN)	AN-specific
(Kinnaird et al. (14)) *Eating as an autistic adult*	Eating behaviours (including participants with AN, BN and BED)	Disordered eating
(Brede et al. (17))	Anorexia nervosa (AN)	AN-specific
(Healy et al. (31))	Weight management, body image and obesity	Disordered eating
(Babb et al. (28))	Anorexia nervosa (AN)	AN-specific
(Sedgewick et al. (38))	Eating disorder recovery across diagnoses	Multiple eating disorders
(Nimbley et al. (37))	Anorexia nervosa (AN)	AN-specific
(Creese et al. (29))	Anorexia nervosa (AN)	AN-specific
(Downs & Adams (30))	Severe eating disorders (predominantly AN/ARFID discussed)	Multiple eating disorders
(Li et al. (33))	Eating disorder inpatient services	Multiple eating disorders
(Nimbley et al. (36))	Research priorities for autistic people with eating disorders	Multiple eating disorders
(Makin et al. (34))	Binge Eating Disorder (BED)	BED-specific
(McIntosh & Hunter (35))	Recovery from anorexia nervosa	AN-specific

**Table 4:** Critical appraisal skills programme (CASP) qualitative checklist quality assessment of included studies.

Study	CASP score (out of 10)	Quality rating
(Babb et al. (28))	10	High
(Brede et al. (17))	9	High
(Creese et al. (29))	9	High
(Downs and Adams (30))*	6	Moderate
(Healy et al. (31))	9	High
(Kinnaird et al. (14))	10	High
(Li et al. (33))	8	High
(Makin et al. (34))	10	High
(McIntosh and Hunter (35))	9	High
(Nimbley et al. (36))	10	High
(Nimbley et al. (37))	10	High
(Sedgewick et al. (38))	6	Moderate
(Kinnaird et al. (14, Same Behaviours))	9	High

*this was an autoethnography which may have affected CASP scoring.

Positionality Statement:

BW, the primary author of this review and the researcher responsible for conducting the thematic synthesis, generating codes, and developing themes, does not identify as neurodivergent and has no personal lived experience of an eating disorder. This position was acknowledged throughout the analytic process, with reflexive consideration given to how the researcher’s perspectives and assumptions may have influenced the interpretation of the data.

Eleven of the 13 studies were rated as high quality and 2 were rated as moderate quality, with no studies classified as low quality. Studies meeting 8–10 CASP criteria were classified as high quality, whereas studies meeting 5–7 criteria were classified as moderate quality. The strongest methodological areas were the appropriateness of the qualitative methodology, consideration of ethical issues, and the value of the research, all of which were met by all 13 studies. Most studies also demonstrated clear research aims, appropriate research designs and recruitment strategies, adequate data collection methods, and clear statements of findings. The most commonly identified limitation was researcher reflexivity, with only 7 studies adequately considering the relationship between the researcher and participants, while 6 studies failed to address this. In addition, three studies did not provide sufficient evidence of rigorous data analysis. Despite these limitations, the overall quality of the evidence base was considered strong, with the majority of studies meeting most or all CASP criteria and providing credible and relevant findings for inclusion in the synthesis.

## Results

Thirteen studies met the inclusion criteria and were included in the thematic synthesis. Application of the three-stage thematic synthesis approach described by Thomas and Harden (25) resulted in the development of 5 descriptive themes and 3 subsequent analytical themes which captured autistic adults’ experiences of eating disorders. The themes identified across studies represent common patterns within participants’ accounts. Each analytical theme is presented below, supported by evidence from the included studies. Quotes were only selected from autistic participants and not from parents or professionals to ensure the voices represented are of those who experience the intersection of identities. Some of the studies included offered multiple perspectives and line by line coding was only carried out on the relevant quotes. Additionally, one paper discussed neurodivergence more broadly, and therefore where applicable, quotes were taken from autistic participants rather than solely those with ADHD. Three of the included studies explored the lived experiences of women exclusively, while the remaining 10 recruited participants of multiple genders. However, female participants constituted the majority of these mixed-gender samples, meaning that the synthesis was informed predominantly by the experiences of women.

Across the included studies, the chronological relationship between identifying the eating disorder and autism was not consistently reported. However, five studies described participants experiencing eating difficulties, accessing eating disorder services, or receiving an eating disorder diagnosis prior to being identified as autistic (14, 17, 28–30), with autism frequently recognised later in adulthood. Autism identification subsequently provided some participants with a framework through which to retrospectively understand their eating disorder experiences.

*Theme 1: Recovery is enabled through the development of self-understanding and the integration of autism into personal identity*.

The first analytical theme was developed from two descriptive themes: “*Understanding Autism as a Framework for Self-Understanding”* and “*Recovery as Identity Development, Connection and Adaptation”.* Across studies, participants described recovery as a process of making sense of their experiences through an autism-informed lens. Rather than viewing their difficulties as personal failings, individuals reported that understanding their autistic identity helped them to reinterpret longstanding challenges, develop greater self-compassion, and build a more coherent sense of identity.

Participants frequently described spending many years searching for explanations for their experiences before having the knowledge and understanding that they were autistic. One autistic individual reflected on a persistent sense of uncertainty, describing “*not knowing why I found it so difficult to do things that other people seemingly were able to join in with*” (30). Similarly, participants in Makin et al. (34) described recognising previously unexplained links between autism and eating difficulties, with one autistic individual noting that there were “*a lot of explanations that were applicable*”. Developing this understanding appeared to facilitate recovery by helping individuals make sense of behaviours that had previously felt confusing. As one autistic individual explained, “*once you understand the reasons that you do stuff, then it’s much, much easier to change your behaviour*” (34).

Beyond increasing understanding of their eating difficulties, participants described understanding autism and how they relate to it as a door to fostering greater self-acceptance. Several accounts suggested that recognising the influence of autism reduced self-blame and enabled individuals to view their difficulties more compassionately. This was particularly highlighted among participants who reflected on how earlier identification may have altered their experiences. One autistic individual stated, “*If only I’d known different, I could have possibly managed this with some adaptions … I possibly wouldn’t have been so hard on myself*” (29). These findings suggest that understanding autism allowed participants to reinterpret previous struggles within the context of neurodivergence rather than personal inadequacy. Alongside increased self-understanding, receiving an autism diagnosis could also bring more complex emotional responses. For some participants, retrospectively reinterpreting their experiences appeared to involve a potential form of grief around what could have been different had their autistic needs been recognised earlier. Downs and Adams (30), for example, described the diagnostic process as both providing clarity and prompting grief for previously misunderstood aspects of the self and missed opportunities.

Recovery was also discussed within the development of identity, connection, and belonging. Participants described feeling isolated prior to understanding that they were autistic and valued opportunities to connect with others who shared similar experiences. One autistic individual reported that being aware that others also experienced both autism and eating difficulties “*made me feel less alone because then I realised that here are other people who also have both [ED and autism], and other people have similar experiences*” (29). These connections appeared to support identity development and foster a sense of acceptance beyond the eating disorder itself.

Taken together, the studies suggest that recovery involved more than symptom reduction and recovery was facilitated through a process of developing an autism-informed understanding of self. This enabled participants to make sense of their experiences, challenge self-critical narratives, and cultivate a more positive and coherent identity. Connection with others who shared similar experiences further reinforced this process, highlighting the importance of self-understanding and a sense of belonging as foundations for recovery.

Theme 2: *Eating Disorder Experiences Cannot Be Understood Independently of Autistic Ways of Processing the World*.

This analytical theme was developed from the descriptive themes “*Understanding Autism as a Framework for Self-Understanding*” and “*Autism Characteristics Shape Eating Disorder Experiences and Recovery*”. Across studies, participants described how developing an understanding of their autism enabled them to recognise the ways in which autistic characteristics influenced their eating difficulties. Sensory sensitivities, interoceptive differences, cognitive profiles, and a preference for routine and predictability were frequently identified as shaping both the development and maintenance of eating disorder behaviours. Participants reported that recognising these connections helped them make sense of their experiences and informed more effective and personalised approaches to recovery.

Participants frequently described sensory experiences as central to their relationship with food and eating. Food choices and eating behaviours were often influenced by sensory preferences and aversions rather than concerns relating to body image or weight. One autistic individual explained that weight gain facilitated food restriction because they disliked the associated bodily sensations, stating, “*I don’t like the sensation of how my stomach feels”* (17). Sensory sensitivities also extended beyond food itself, with participants describing environmental factors as barriers to eating. For example, one autistic individual attributed the onset of food restriction during adolescence to the overwhelming sensory demands of the school environment, describing “*a big canteen, lots of people, lots of social stuff going on, lots of noise”* (17). These accounts suggest that sensory experiences often played a significant role in shaping eating behaviours and maintaining eating difficulties.

Participants also reported difficulties recognising and interpreting internal bodily signals, which influenced their ability to regulate their food intake. Many described uncertainties around hunger, fullness, thirst, and satiety, resulting in both under and overeating. One autistic individual explained, “*I don’t really fully understand my thirst and hunger responses, or my fullness responses, so that really influences my eating because I can binge or miss meals very, very easily”* (17). Similarly, participants in Nimbley et al. (37) described recognising physical sensations such as dizziness, nausea, or stomach discomfort without identifying them as indicators of hunger. Developing an understanding of these interoceptive differences and signals enabled some participants to reframe their eating difficulties as arising from autistic experiences rather than a lack of motivation or self-control.

Cognitive profiles associated with autism were also frequently described as influencing eating disorder experiences. Participants described a strong preference for predictability and consistency, which influenced their eating behaviours. One autistic individual explained that “*the rules we tend to make or create in our minds … become so important to us … the rules cannot be broken”* (36). Similarly, participants described becoming entrenched in eating-related routines that were difficult to move away from, with one autistic individual stating, “*I’m a creature of habit and I get into patterns with food that are generally bad patterns”* (31). While these characteristics could contribute to the maintenance of eating difficulties, participants also reported that structured and predictable approaches to recovery could be beneficial. For example, one autistic individual described how “*making meal plans helps”* by reducing uncertainty and supporting engagement with eating (14).

Importantly, participants often linked these autistic characteristics directly to the development and persistence of their eating difficulties. One autistic individual reflected that “*the autism and the routine and rigid thinking maintained the eating disorder”* and concluded that “*for me, the anorexia is just a symptom, and the cause is the autism”* (17). Across studies, participants described gaining a greater understanding of these relationships through autism identification and psychoeducation. This understanding helped individuals contextualise their experiences and challenged explanations that framed eating difficulties solely in terms of body image concerns or psychopathology.

Overall, the findings suggest that eating disorder phenomenology cannot be understood independently of the autistic experience of processing and navigating the world. Participants consistently described eating difficulties as being shaped by sensory experiences, interoceptive differences, differences in cognitive profiles and a preference for predictability. Understanding the relationship between autism and eating difficulties enabled individuals to reinterpret behaviours that had previously felt confusing or self-defeating and informed more personalised approaches to recovery. The synthesis therefore indicates that recognising the influence of autism is central not only to understanding eating disorder experiences but also to supporting meaningful and sustainable recovery.

Theme 3: *Recovery is Facilitated When Support Aligns with Autistic Needs Rather Than Neurotypical Assumptions*.

This analytical theme was developed from the descriptive themes *Feeling Misunderstood and Overlooked by Others and Services* and *Recovery Requires Individualised and Autism-Informed Support.* Across studies, participants described how recovery was often hindered when professionals interpreted their experiences through conventional eating disorder frameworks that failed to recognise the influence of autism. Participants frequently reported feeling misunderstood, dismissed, or required to explain their needs repeatedly. In contrast, support was experienced as helpful when practitioners recognised the interaction between autism and eating difficulties, adapted their approaches accordingly, and prioritised individual needs over standardised treatment models.

Many participants described experiences of being misunderstood by professionals, particularly when autistic characteristics were interpreted solely through the lens of an eating disorder. One autistic individual recalled that clinicians viewed long-standing autistic behaviours as symptoms of anorexia, explaining, “*When I was in hospital, I kept getting told off for walking on tip toes and for fidgeting a lot … they thought I was doing these things to burn more calories, except I’d been doing them for as long as I could remember”* (28). Similarly, participants reported that their difficulties were frequently attributed to eating disorder pathology even when alternative explanations were offered. One autistic individual explained that “*Nobody really acknowledged my autism for a few months … and they were like ‘nah that’s just your eating disorder’*” (35). These experiences often left individuals feeling invalidated and unsupported.

Participants also described how assumptions embedded within traditional eating disorder models could be unhelpful and, in some cases, harmful. Several accounts highlighted frustration when clinicians assumed that body image concerns were the primary driver of eating difficulties. One autistic individual explained, “*She basically didn’t believe me when I said this is not about [body image] … she wanted to do body image therapies with me and I … found it humiliating, and also irritating and condescending that I was being not listened to”* (28). Likewise, another autistic individual reflected that professionals were “*very focused on whether or not I thought I was overweight”* despite this not being relevant to their experience (35). These findings suggest that neurotypical assumptions about eating disorders often failed to capture the complexity of participants’ experiences and could hinder engagement with treatment.

In contrast, participants consistently valued support that was individualised, collaborative, and responsive to autistic needs. Rather than applying standardised interventions, effective practitioners sought to understand the individual and adapt support accordingly. As one autistic individual explained, “*if you took me and someone else with ARFID, then our treatments will be so incredibly different because of the different routes*” and therefore “*categorising people, not just by their symptoms but by how this has come about, is going to be a lot more effective to treat them*” (38). Participants emphasised that autistic people have diverse needs and that treatment approaches should reflect this diversity. One autistic individual noted that “*Neurodivergent people have different needs [that] might change over time*” (34).

Support was perceived as particularly effective when practitioners demonstrated an understanding of autism and actively accommodated communication preferences, sensory needs, and cognitive styles. One autistic individual described a therapeutic relationship as a “*turning point*” because the therapist “*understood how best to communicate with me”*, providing written summaries and using goal-setting approaches to structure care (29). Similarly, another autistic individual reported that simple adjustments, such as providing advance notice of changes and allowing comforting sensory items, “*can make a really big difference”* (28). Respecting personal boundaries was also viewed as important, with one autistic individual explaining that family members recognised their “*‘nope’ reflex”* and did not pressure them to engage with foods that felt intolerable, which “*helps tremendously”* (14).

Across studies, participants highlighted the importance of feeling seen as a person rather than as a diagnosis. One autistic individual reflected, “*We try to categorise things so much we lose sight of the person, and I think it’s just the person we need to be looking at”* (38). Others emphasised the value of having their experiences recognised as legitimate, with one autistic individual stating, “*My experiences, be they atypical, are still perceived as valid”* (14). These accounts suggest that effective support was characterised not only by autism-specific adaptations but also by respect, validation, and genuine collaboration.

The findings suggest that recovery is facilitated when support moves beyond neurotypical assumptions and instead aligns with autistic ways of experiencing the world. Participants consistently described difficulties when services prioritised conventional eating disorder models over individual experiences, often resulting in misunderstanding, invalidation, and inappropriate interventions. Conversely, recovery was supported when practitioners recognised the intersection of autism and eating difficulties, adapted treatment to individual needs, and worked collaboratively to identify meaningful and achievable goals. The synthesis therefore indicates that autism-informed, person-centred support is not simply a preference but a fundamental component of effective recovery-oriented care.

## Discussion

This systematic review provides a synthesis of the lived experience of autistic adults with eating disorders and disordered eating. Throughout the evidence base, there has been an established association between autism and eating disorders; however, the current synthesis extends this by outlining that developing an understanding of one’s autistic identity fundamentally altered how individuals understood their eating-related difficulties. Our findings highlight that recovery can be underpinned by a process of reframing eating disorder experiences through an autism-informed lens. Rather than simply having more knowledge about autism, having this understanding enabled individuals to reframe and reinterpret their experiences that were once seen as personal failings or solely related to eating disorder psychopathology. Viewing this through an autism-informed lens, their experiences can be seen as understandable responses to autistic ways of processing and navigating the world. Being able to shift their perspective in this way appeared to facilitate greater self-compassion, promote recovery through a more person-centred and autism-informed way, and support the development of a more coherent autistic identity. However, this process of reinterpretation was not solely positive, with some participants describing a potential sense of grief around what could have been different had their autism been recognised earlier. This is consistent with wider qualitative literature on late autism diagnosis, which suggests that increased self-understanding can coexist with grief associated with revisiting previously misunderstood experiences and considering how life may have differed with earlier recognition (40, 41).

The findings of the current synthesis suggest that conventional eating disorder models may not completely capture autistic experiences because they tend to highlight disturbances with body image as a primary explanatory mechanism. Jointly, the findings across the papers synthesised suggest that eating disorders and autism should not be conceptualised solely in terms of co-occurring diagnoses but as reflecting the ways in which autistic experiences fundamentally shape how eating disorders are experienced, interpreted, and responded to across personal and clinical contexts. This highlights the importance of understanding the lived phenomenology of autistic adults with eating disorders, rather than conceptualising autism and eating disorders simply as co-occurring conditions. Rather, autistic individuals who participated in the studies described eating-related difficulties as embedded within autistic experiences that may represent different maintaining processes, such as sensory processing, interoception, and a preference for predictability. Participants frequently described restrictive eating as driven by sensory experiences rather than concerns about weight or shape, suggesting that autistic eating difficulties may not always align neatly with conventional diagnostic criteria. It should be highlighted that the evidence base predominantly focused on anorexia nervosa. Although ARFID was formally recognised during the period in which these studies were published, increasing recognition of ARFID within clinical practice has been relatively recent. Consequently, it is possible that some autistic individuals whose eating difficulties were primarily characterised by sensory sensitivities or food avoidance may historically have been conceptualised within anorexia nervosa. However, this raises a broader question regarding the distinction between ARFID and restrictive eating associated with autism. ARFID and autism frequently co-occur and share features relating to sensory sensitivities, food preferences and restrictive eating patterns (42, 43). However, restrictive eating associated with autism should not necessarily be equated with ARFID, as feeding difficulties may occur without meeting criteria for a separate ARFID diagnosis (44). The extent to which ARFID represents a distinct co-occurring disorder or whether some presentations reflect eating behaviours embedded within autistic sensory and behavioural experiences remains an area requiring further investigation (43). The present synthesis cannot resolve this distinction but highlights the importance of understanding the function and phenomenology underlying restrictive eating rather than relying on behavioural presentation and categorical diagnosis alone.

Together, these findings have implications for how eating disorders are conceptualised, diagnosed and formulated within autistic populations. For example, Fairburn, Cooper and Shafran’s (45) Transdiagnostic Cognitive Behavioural Model emphasises the overevaluation of weight and shape whilst also maintaining cognitive and behavioural processes, while Schmidt and Treasure’s (46) Cognitive Interpersonal Maintenance Model extends this by recognising the contribution of emotional processing, interpersonal factors and differences in cognitive profiles, factors that are more adjacent to autism. These findings therefore extend existing cognitive models, rather than reject them completely, by suggesting that autistic characteristics may shape both the presentation of eating disorders and the functions that eating-related behaviours serve. In doing so, the findings align with neurodiversity-informed perspectives by positioning autism as a different way of experiencing and interpreting the world rather than a co-occurring disorder. The synthesis also resonates with personal recovery theories, such as the CHIME Framework (47), which conceptualises recovery as a process of reconstructing identity and meaning. However, the present synthesis extends these frameworks by suggesting that, for autistic adults with an eating disorder, identity reconstruction may be preceded by re-framing experiences that were previously understood as personal failures or understanding their eating disorder through an autism-informed understanding of themselves. The present synthesis therefore suggests that autism-informed sense-making may be a previously under-recognised process through which autistic adults reinterpret eating disorder experiences, develop a more coherent sense of self, and engage more meaningfully with recovery. Rather than replacing existing eating disorder or recovery models, this interpretation complements them by identifying a process that may be particularly salient within autistic populations.

There are several methodological strengths that contribute to the reliability of the findings. Firstly, the review conducted a systematic search across four databases alongside manual reference list checking which reduces the likelihood of missing relevant studies and increasing the comprehensiveness of the evidence base (24). Additionally, adherence to PRISMA reporting guidelines promotes methodological transparency and reproducibility. Conducting a thematic synthesis (25) is a further strength as this facilitates the generation of analytical themes whilst staying close to the participants’ lived experiences. The review also deliberately focused on first-person accounts from autistic adults instead of perspectives of parents, carers or clinicians which reflects neurodiversity informed research principles. Finally, the study is further strengthened by the methodological quality of the included studies, with the majority meeting a high standard on the Critical Appraisal Skills Programme (CASP) checklist. Overall, the decisions made within this review increase confidence that the analytical themes identified represent meaningful patterns across the existing and available qualitative evidence base.

Despite these strengths, several limitations should be considered when interpreting the findings of this review. The review search was restricted to papers published in English-language that have undergone a peer review process. This introduces a possibility for language and publication bias, which may exclude relevant studies of unpublished qualitative findings or studies conducted in non-English speaking countries or populations. Although qualitative synthesis provides depth in understanding rather than producing generalisable findings, the transferability of the present synthesis should be considered in light of the available evidence base. Only 13 studies met the inclusion criteria, and much of the literature focused on autistic adults with anorexia nervosa, with limited representation of other eating disorder diagnoses and demographic groups. Given the heterogeneity within both autism and eating disorders, it cannot be assumed that the analytical themes fully capture the breadth of experiences across the wider autistic population. Consequently, the findings should be interpreted as representing common patterns within the included studies rather than universal characteristics of autistic adults with eating disorders. Although consideration of sex and gender differences was beyond the scope of the present review, the included studies predominantly reflected the experiences of women. Future qualitative research should seek to include greater representation of participants of different genders to explore whether the lived experience of autism and eating disorders varies according to sex or gender. Finally, despite methodological quality ranking highly, the CASP quality appraisal identified that researcher reflexivity was the most common methodological weakness, with almost half of the included studies failing to adequately consider how researchers’ assumptions, positions, and relationships may have influenced data collection or interpretation. Reflexivity in qualitative research is fundamental to establishing rigor, and therefore this limitation should be held in mind when evaluating the credibility of the findings.

Moving forward, future research should continue to move beyond categorical diagnostic labels and instead focus on the underlying drivers and mechanisms through which autistic experiences shape and maintain eating related difficulties and disorders. The current evidence base focuses mainly on the experiences of individuals with anorexia nervosa and little on those who experience conditions such as bulimia nervosa, binge eating disorder or ARFID. Given that within this synthesis sensory sensitivities and interoceptive differences have been highlighted as common experiences, future studies should explore whether these experiences represent mechanisms that are underlying across different eating disorder presentations. In particular, the findings highlight important conceptual links with ARFID, where sensory sensitivities, reduced interoceptive awareness, and avoidance of food based on sensory characteristics are central features. Participants frequently described restrictive eating as driven by sensory experiences rather than concerns about weight or shape, suggesting that autistic eating difficulties may not always align neatly with conventional diagnostic criteria. This is important for clinicians and researchers to be aware of to avoid misdiagnosis or a missed diagnosis and to also ensure eating disorder research fully captures the voices of all, rather than a sub-group. Further research is therefore needed to understand how sensory processing contributes to different eating disorder presentations in autistic adults and whether existing diagnostic frameworks appropriately capture these experiences.

The findings of this synthesis also have important implications for clinical practice. Assessment should extend beyond traditional eating disorder formulations to routinely consider the functional role that eating behaviours serve for autistic individuals. Clinicians should adopt collaborative, autism-informed formulations that recognise the diversity of autistic experiences rather than assuming that all eating disorders are solely focused on body image or weight concerns. Interventions should incorporate reasonable adjustments such as predictable treatment structures, sensory adaptations, flexible communication methods, and psychoeducation that supports individuals to understand the relationship between autism and their eating difficulties. Although recognising the co-occurrence of autism and eating disorders is important, the findings of this synthesis suggest that co-occurrence alone may not fully capture the lived phenomenology of autistic adults with eating disorders. Rather, autism fundamentally shapes how eating disorders are experienced, understood, and recovered from. Developing an understanding of this lived experience may therefore be important for informing autism-informed formulations and providing more personalised support that promotes meaningful engagement and recovery.

## Data Availability

The original contributions presented in the study are included in the article/supplementary material. Further inquiries can be directed to the corresponding authors.
